# Sensitive liquid biopsy monitoring correlates with outcome in the prospective international GPOH-DCOG high-risk neuroblastoma RT-qPCR validation study

**DOI:** 10.1186/s13046-024-03261-y

**Published:** 2024-12-26

**Authors:** Lieke M. J. van Zogchel, Boris Decarolis, Esther M. van Wezel, Lily Zappeij‐Kannegieter, Nina U. Gelineau, Roswitha Schumacher‐Kuckelkorn, Thorsten Simon, Frank Berthold, Max M. van Noesel, Marta Fiocco, C. Ellen van der Schoot, Barbara Hero, Janine Stutterheim, Godelieve A. M. Tytgat

**Affiliations:** 1 Princess Máxima Center for Pediatric Oncology, Utrecht, The Netherlands; 2 Department of Experimental Immunohematology, Sanquin Research and Landsteiner Laboratory of the Amsterdam UMC, Amsterdam, The Netherlands; 3 Department of Pediatric Oncology and Hematology, University Children’s Hospital of Cologne, and Medical Faculty, University of Cologne, Köln, Germany; 4 Department of Immunocytology, Sanquin Research and Landsteiner Laboratory of the Amsterdam UMC, Amsterdam, The Netherlands; 5 Division Imaging & Oncology, University Medical Center Utrecht, Utrecht, the Netherlands; 6 Mathematical Institute, Leiden University, Leiden, The Netherlands; 7 Department of Biomedical data Science, Section Medical Statistics, Leiden University Medical Center, Leiden, The Netherlands; 8 Department of Genetics, Utrecht University Medical Center, Utrecht, the Netherlands

## Abstract

**Background:**

Liquid biopsies offer less burdensome sensitive disease monitoring. Bone marrow (BM) metastases, common in various cancers including neuroblastoma, is associated with poor outcomes. In pediatric high-risk neuroblastoma most patients initially respond to treatment, but in the majority the disease recurs with only 40% long-term survivors, stressing the need for more sensitive detection of disseminated disease during therapy.

**Methods:**

To validate sensitive neuroblastoma mRNA RT-qPCR BM testing, we prospectively assessed serial BM samples from 345 international high‐risk neuroblastoma patients, treated in trials NB2004 (GPOH) or NBL2009 (DCOG), using *PHOX2B*, *TH*, *DDC*, *CHRNA3*, and *GAP43* RT-qPCR mRNA markers and BM GD2-immunocytology. Association between BM-infiltration levels and event-free survival (EFS) and overall survival (OS) was estimated by using Cox regression models and Kaplan-Meier’s methodology.

**Results:**

BM infiltration >10% by RT-qPCR at diagnosis was prognostic for survival (adjusted hazard ratio (HR) 1.82 [95%CI 1.25‐2.63] and 2.04 [1.33‐3.14] for EFS and OS, respectively). Any post-induction RT-qPCR positivity correlated with poor EFS and OS, with a HR of 2.10 [1.27-3.49] and 1.76 [1.01-3.08] and 5-years EFS of 26.6% [standard error 5.2%] versus 60.4% [6.7] and OS of 43.8% [5.9] versus 65.7% [6.6] for RT-qPCR-positive patients versus RT-qPCR-negative patients. In contrast, post-induction immunocytology positivity was not associated with EFS or OS (HR 1.22 [0.68-2.19] and 1.26 [0.54-2.42]).

**Conclusion:**

This study validates the association of not clearing of BM metastases by sensitive RT-qPCR detection with very poor outcome. We therefore propose implementation of RT-qPCR for minimal residual disease testing in neuroblastoma to guide therapy.

**Supplementary Information:**

The online version contains supplementary material available at 10.1186/s13046-024-03261-y.

## Background

Liquid biopsies have shown promise in assisting diagnosis and monitoring therapy response in adult oncology [1] and have been implemented into clinical practice over the past years. The most widely adopted and investigated source for liquid biopsies is blood, although the term also comprises other body fluids such as urine, cerebrospinal fluid and bone marrow (BM). BM is a preferred site of metastatic disease in various adult and pediatric cancers, such as breast cancer, prostate cancer, and neuroblastoma [2]. Consequently, the monitoring of BM disease is pivotal to assess therapy response and disease recurrence. Neuroblastoma, the most common pediatric extracranial solid tumor has a broad spectrum of clinical behavior, ranging from spontaneous regression to incurable aggressive disease [3]. One of the most powerful predictors of outcome in patients with neuroblastoma is metastatic disease [4, 5]. BM is the most common site of metastatic disease at diagnosis [4, 6] and a frequent site of relapse [7]. In current clinical practice, assessment of treatment response is based on the International Neuroblastoma Response Criteria (INRC): Meta-iodobenzylguanidine (MIBG) scintigraphy, MRI, CT scans, PET scans, and BM examinations by histology or (immuno)cytology are combined to assess the extent of disease [8]. However, with current assessments, about 50% of all patients in presumed complete remission will relapse, leading to only 40% long term survivors [9, 10]. The prognosis of patients with recurrent or refractory neuroblastoma is dismal with 4-year progression free survival and overall survival (OS) of 6% and 20%, respectively [11]. This underlines the urgent need for more sensitive techniques to establish which patient will or will not be cured by the current therapies, and thus to stratify patients in function of response. As metastatic response to induction therapy is associated with outcome, at designated time points, bone marrow is tested for metastases by histology, cytology and immunocytology. Histology and cytology have a restricted sensitivity, can be more difficult to standardize and could therefore underestimate the BM infiltration during treatment [12]. Immunocytology is a more sensitive, standardized technique, and is implemented in several clinical protocols, but is time-consuming and requires experienced investigators [13, 14]. Reverse transcriptase quantitative polymerase chain reaction (RT-qPCR) provides highly sensitive means of detecting minimal residual disease (MRD) in BM and peripheral blood (PB) [5, 15]. In leukemia, MRD detection by (RT-)qPCR is a highly validated, inexpensive, easily standardized technique that has been implemented two decades ago and is performed worldwide to guide therapeutic decisions [16]. In neuroblastoma, detection of mRNA in BM by RT-qPCR during treatment has also been shown to be of prognostic value and a predictor of poor outcome [15, 17–19]. We were the first to describe paired-like homeobox 2b (*PHOX2B*) as a highly sensitive and neuroblastoma specific mRNA marker for MRD detection [20]. Yet, as *PHOX2B* expression levels vary between tumors, we further showed that adding other markers, tyrosine hydroxylase (*TH*), dopamine decarboxylase (*DDC*), cholinergic receptor nicotinic alpha 3 (*CHRNA3*), and growth associated protein 43 (*GAP43*), contributes to more sensitive MRD detection [21, 22] and the clinical utility for patients with neuroblastoma of all risk groups [15, 23, 24]. In a large prospective study of the European Society of Pediatric Oncology Neuroblastoma Group (SIOPEN), Viprey et al. showed in BM of high-risk patients, transcripts of *TH*, *PHOX2B*, or doublecortin (*DCX*) above a certain threshold, defined in a training cohort, to correlate with a poorer prognosis than patients showing no or lower levels of BM infiltration [17]. These thresholds were based on therapy responses, potentially requiring validation in different treatment regimens. In a retrospective study in patients with high-risk neuroblastoma, we demonstrated the prognostic value of our mRNA panel at diagnosis, and the significance of fast BM clearance [15]. In the study presented here, we prospectively validate these findings in an international cohort of children with high-risk neuroblastoma treated according to high-risk protocols NB2004 (GPOH) or NBL2009 (DCOG). The clinical significance of BM infiltration levels at diagnosis and clearing of the BM after induction therapy is measured by sensitive RT-qPCR for a neuroblastoma-specific mRNA panel and anti-GD2 immunocytology.

## Methods

### Patients and samples

Patients were included in this prospective study if they were (a) diagnosed with high-risk neuroblastoma between 2009 and 2017, (b) treated according to the German GPOH NB2004-HR trial [10] or the Dutch DCOG NBL2009 trial [25] (Supplemental Figure 1), and (c) if written informed consent from parents or guardians was obtained according to the declaration of Helsinki. Patients within the German GPOH NB2004-HR trial were randomized to receive prolonged induction therapy, but Berthold and colleagues reported equal outcomes for EFS and OS [10]. Within the high-risk protocol, standard induction therapy consisted of six alternating courses of N5 (vindesine, cisplatin, etoposide) and N6 (vincristine, dacarbacine, ifosfamide, doxorubicin), (identical in both GPOH and DCOG trials), which – depending on randomization results- were eventually preceded by, two additional courses of topotecan, cyclophosphamide and etoposide (N8) in the GPOH trial [10], or if clinically achievable, upfront 2 courses of MIBG therapy in the DCOG trial [25]. After induction therapy, patients received high dose chemotherapy with autologous hematopoietic stem cell rescue and isotretinoin for consolidation. Dutch patients diagnosed after 2012 (if eligible; patients in complete- or very good partial remission) received GD2 immunotherapy in the Children’s Hospital Philadelphia, USA [26]. For German patients, immunotherapy was not scheduled per protocol, but given to single patients after 2010.

Diagnostics and staging procedures were performed according to the International Neuroblastoma Staging System (INSS) [27, 28]. High-risk patients were defined as stage 4 over 1 year of age or all stages with *MYCN* amplification. The study was approved by the Medical Research Ethics Committees of the Academic Medical Center (Amsterdam, the Netherlands; MEC07/219#08.17.0836) and the University of Cologne (Cologne, Germany).

BM aspirates from two to four sites were collected in EDTA tubes at diagnosis and at dedicated time points during induction chemotherapy: after first 2 therapy courses (after 2x N8/ 2x MIBG therapy/ or the first N5/N6), and at the intended end of induction (Supplemental Figure 1). In Germany, bilateral BM samples were pooled prior to processing, the Dutch bilateral BM samples were analyzed individually by RT-qPCR. BM samples were transferred to PAXgene blood RNA tubes (QIAGEN, Venlo, the Netherlands) within 24 hours and then stored at -20°C. RNA isolation and RT-qPCR were performed in Amsterdam, the Netherlands, as described below.

### RNA extraction and RT-qPCR

RNA was isolated from PAXgene blood RNA tubes (QIAGEN, Venlo, Netherlands) with the PAXgene Blood RNA Kit (QIAGEN). cDNA was synthesized from 2-3 µg of RNA, using 25 µmol/L random hexamers (Invitrogen, Carlsbad, CA, USA), 1 mmol/L dNTPs (Promega, Madison, WI, USA) and 100U of MMLV transcriptase (Invitrogen, ThermoFisher, Waltham, USA), in a total reaction volume of 40 µl and incubated at 42°C for 45 minutes. Finally, the reverse transcriptase was inactivated by heating and the volume was diluted to 100 µl. RT-qPCR for *PHOX2B*, *TH*, *DDC*, *CHRNA3* and *GAP43* was performed using beta-glucoronidase (*GUSB*) for normalization [21] on Step-One-Plus or Viia7 (Applied Biosystems, Carlsbad, CA, USA). Primers and probes sequences have been published previously [21, 29] and were synthesized by Eurogentec (Liege, Belgium). Reactions were carried out in 20 µL (10 µL TaqMan^TM^ Fast Universal PCR Master Mix (Applied Biosystems), 300 nM forward and reverse primer and 200 nM probe and 5 µL cDNA). In all RT-qPCR reactions, initial heating was done for 20 s at 95 °C, followed by 50 cycles of 1 s at 95 °C and 20 s at 60 °C. All RT-qPCR experiments were carried out in triplicate (except *GUSB*, which was performed in duplicate) and mean values were used for analysis. All samples with a Ct for *GUSB* >25 were excluded. Marker expression was normalized to *GUSB* expression using the following equation [(ΔCt) = Ctmarker – Ct*GUSB*]. In short, PHOX2B was considered positive if there was any amplification, as the PHOX2B used by our lab has no background expression [20]; the other markers were considered positive if the Ct was <40 and ΔCt of the sample was <3 Ct than the ΔCt of the normal control BM samples, as described previously [21]. A sample was scored positive if the Ct of at least one out of five markers, was above the threshold for positivity, as has been described previously, determined in 51 pediatric BM samples [21]. To estimate the level of infiltration, the expression level of the mRNA RT-qPCR targets were related to the expression level of an external standard (neuroblastoma cell line IMR-32) according to the following formula: 2^-ΔΔCt (ΔCt sample - ΔCt IMR-32) * 100%. IMR-32 is one of the most frequently investigated cell lines in neuroblastoma research, elaborately tested for stability of our RT-qPCR markers [22] and used across SIOPEN laboratories for quality control and relative quantification [17, 30]. The median relative expression of the markers was used to calculate the level of infiltration of each individual patient/time point. Because Dutch BM samples were not pooled, results were averaged, and in case one site was negative for a marker(s) the positive quantity for that/those marker(s) was halved. If the adjusted marker scored above the threshold, the sample was regarded positive for this marker.

### GD2-immunocytology

For both the German and Dutch patients, immunocytology was carried out in Cologne, Germany, according to the internationally standardized protocols [5, 13, 31, 32]. BM samples, collected in EDTA tubes, from two to four sites were pooled and mononuclear cells were isolated by density gradient centrifugation. Cytospins were stained using the alkaline phosphatase anti-alkaline phosphatase -method. A minimum of one million cells were investigated. Results were given in the categories negative / <1% / 1-10% / 10-30% / 30-100% as estimated by the investigator (R.S.-K.).

### Statistics

Kaplan-Meier’s methodology was used to estimate overall survival (OS) and event-free-survival (EFS, where event was defined as progressive disease, relapse and death) from time since sample acquisition; patients alive were censored at the last follow-up time. To assess the difference between survival outcomes the log-rank test was used. Reverse Kaplan Meier was employed to estimate the median follow-up [33]. A Cox univariable proportional hazards regression model was employed to quantify the effect of prognostics factors on survival outcomes. In addition, multivariable Cox models were estimated including MYCN amplification and age at diagnosis and information about relapse or progressive disease known at the landmark time in addition to RT-qPCR or immunocytology results [34]. A landmark analysis [34] with 2 landmarks points: at diagnosis and after 2 induction courses was employed to estimate the effect of RT-qPCR stratifications and relapse/progressive disease occurred within 2 years from diagnosis on overall survival (OS). In the landmark methodology, a fixed time after diagnosis and after 2 cycles of therapy are selected as landmark point for performing the analysis. Only patients alive at the landmark time are included in each analysis. In the Cox regression model for overall survival, from the landmark point 2 years after diagnosis and after 2 cycles of therapy, information about relapse or progressive disease known at the landmark time were included as risk factor in the model. An interaction term between RT-qPCR and occurrence of relapse/progressive disease known at the starting time of the analysis was also included in the Cox model. All statistical analyses were performed by using SPSS version 26.

## Results

### Patient characteristics

Three hundred forty-five children with high-risk neuroblastoma were included in this study, with a median age at diagnosis of 33.6 months (range 0.3-224 months), see Table 1 for patient characteristics. Patients were treated according to the German GPOH NB2004-HR trial [10] or the Dutch DCOG NBL2009 trial [25] (Supplemental Figure 1). The median follow-up time was 81.5 months [95% CI 76.1-86.9]. In 98% of patients, BM aspirates were available at diagnosis, in 74% after 2 therapy cycles and in 37% at end of induction, respectively (Supplemental Figure 2). We investigated whether previous GD2-immunocytology results (the clinically used standard) influenced sample acquisition. Of the 88 missing bone marrow samples after 2 cycles of therapy, 23 (26%) samples were from patients with a previous sample negative for immunocytology. Of the 257 sampled patients after 2 cycles of therapy, only 30 patients did have a negative previous sample or were not sampled before (12%). Of the 216 patients with missing samples at the end of induction, 102 patients (47%) had a previously negative sample for immunocytology, similarly to the 129 patient that were sampled at the end of induction (61 patients with a previously negative sample; 47%). Numbers at risk and 5-year survival rates for each estimated survival outcomes are presented in Supplemental Table 1.

**Table 1:** Patient characteristics

**Patient characteristics**	
**Country**	
Netherlands	84
Germany	261
**INSS stage**	
2	6
3	19
4	312
4s	7
unknown	1
**Age at diagnosis (months)**	
median	33,6
range	0.3-224.4
**Age at diagnosis**	
<18 months	62
>18 months	283
**Gender**	
Male	214
Female	131
**MYCN**	
Amplification	153
No amplification	188
Unknown	4
**Metastatic disease ánd age >18 months**	
	266
**Allocated treatment**	
Standard arm	222
2x N8 + standard arm	100
MIBG + standard arm	23
**Follow up time (months)**	
Median	81,5
95% confidence interval	76,1-86,9

### Comparison between RT-qPCR and immunocytology

**Fig 1:**
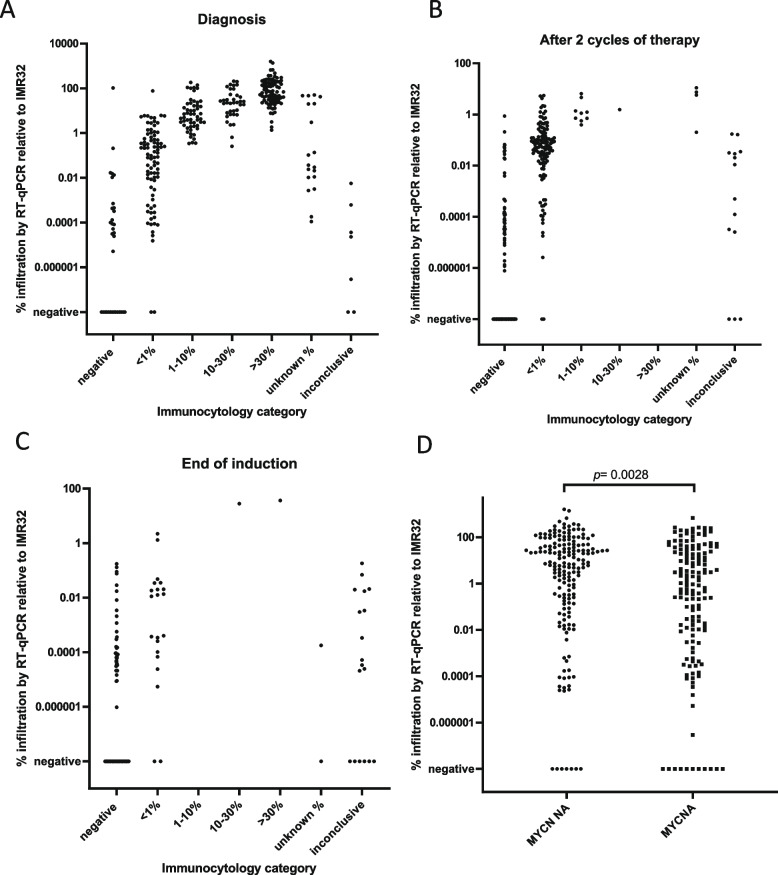
Level of infiltration by RT-qPCR relative to cell line IMR-32 versus GD2-immunocytology at diagnosis (**A**), after 2 cycles of therapy (**B**), end of induction therapy (**C**). **D**. Level of infiltration by RT-qPCR relative to cell line IMR-32 at diagnosis in patients with MYCN non-amplified tumors (MYCN NA) vs MYCN-amplified tumors (MYCNA)

We observed concordance between the percentage BM infiltration estimated by the RT-qPCR mRNA panel and immunocytology in the paired BM samples. For both techniques, the number of positive BM samples and the levels of infiltration were lower after two therapy cycles and at end of induction, compared to the diagnostic samples (Figure 1, Supplemental Figure 3). At all three time-points, RT-qPCR was more sensitive compared to immunocytology, with 14 out 33 (42%), 42 out 86 (49%) and 43 out 80 (54%) RT-qPCR positive/immunocytology negative samples, at diagnosis, after 2 therapy cycles and at end of induction, respectively. At each time-point, only 2 RT-qPCR negative samples were immunocytology positive. At diagnosis, BM mRNA infiltration was significantly lower in BM of patients with MYCN amplified tumors compared to those with MYCN non-amplified tumors, but ranges overlap (median 2.1% [interquartile range 0.02-31.2] versus 10.4% [0.34-51.12], p=0.0028 (Figure 1D).

### Prognostic value of neuroblastoma mRNA level in BM at diagnosis

**Fig 2:**
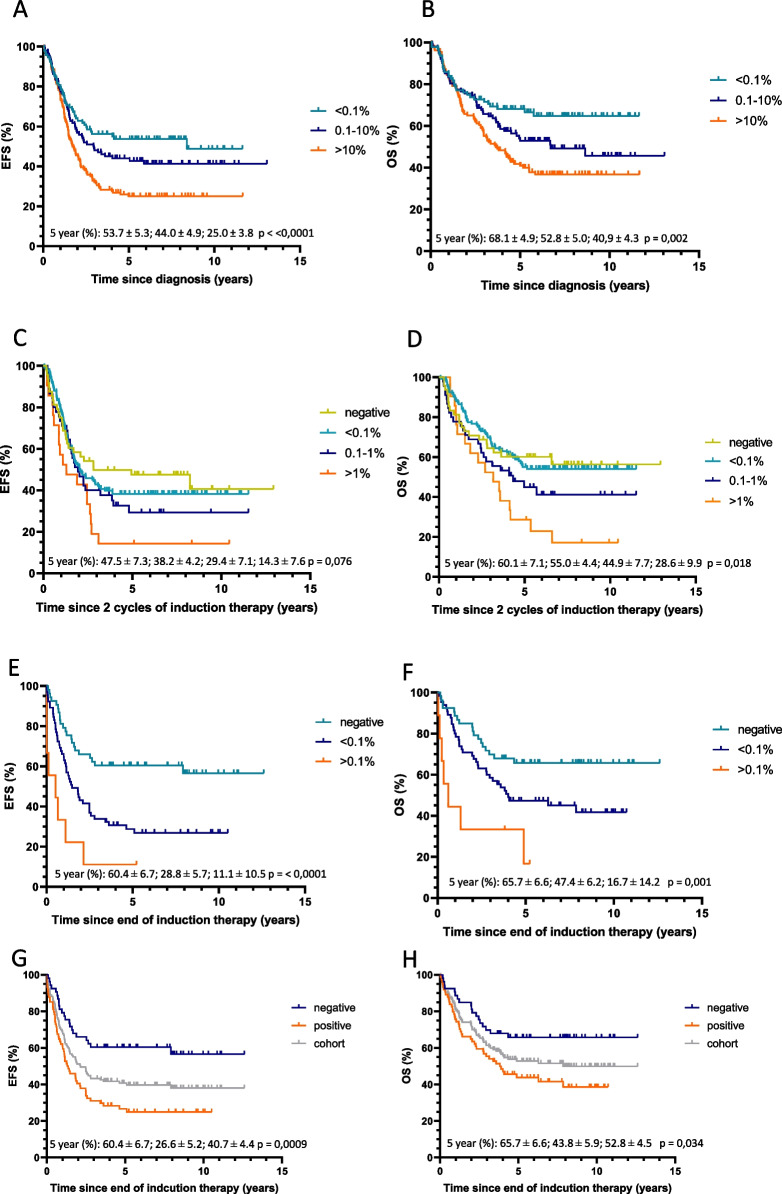
Kaplan-Meier event-free and overall survival curves (EFS on the left and OS on the right, respectively) according to the level of mRNA infiltration by RT-qPCR detected in bone marrow at diagnosis (**A, B**), after 2 cycles of therapy (**C, D**) and after induction therapy (**E, F**). Stratification by RT-qPCR at end of induction compared to the total cohort (**G, H**). 5-year survival rates are given in order according to the legend. The number of children at risk with time for each group is provided in the Data Supplement

At diagnosis, 135 out 329 patients (41%) had high infiltration levels of >10% BM determined by RT-qPCR. These high neuroblastoma mRNA levels are strongly associated with outcome, both EFS and OS in univariable and multivariable analysis: the adjusted hazard ratio (HR) for RT-qPCR group >10% was 1.82 [95% CI 1.25‐2.63] and 2.04 [1.33‐3.14] for EFS and OS, respectively (Figure 2A-B, Table 2), with a 5-year EFS and OS of 25.0% (SE 3.8) and 40.9 (4.3) versus 53.7% (5.3) and 68.1 (4.9) for the <0.1% infiltration group (Supplemental Table 1). The adjusted HR for RT-qPCR infiltration 0.1-10% at diagnosis was 1.29 [0.87-1.93] and 1.52 [0.97-2.40] for EFS and OS, respectively. Also in patients older than 18 months with metastatic disease, HR for RT-qPCR group >10% was significant in multivariable analysis for OS only (Supplemental Figure 4A-B, Table 2). To study the effect of high infiltration levels in the BM at diagnosis after completing therapy, we performed a landmark analysis, with landmark point 2 years after diagnosis. This confirms the prognostic effect of infiltration on overall survival (HR for OS RT-qPCR 0.1-10% 2.42 [1.13-5.19) and RT-qPCR>10% 2.76 [1.32-5.77]), where occurrence of relapse/progressive disease within two years after diagnosis is included in the model as risk factor (Supplemental Figure 5A-C, Table 2).

**Table 2:** Event-free survival (EFS) and overall survival (OS) in Cox proportional hazards regression models

		**Univariable**				**Multivariable (adjusted for MYCN and age >18 months)**			
		**EFS**		**OS**		**EFS**		**OS**	
**Variable**	**patients (No.)**	**HR**	**95% CI**	**HR**	**95% CI**	**HR**	**95% CI**	**HR**	**95% CI**
**Diagnosis, RT-qPCR group**									
<0.1%	92								
0.1-10%	102	1.29	0.87-1.91	1.49	0.95-2.33	1.29	0.87-1.93	1.52	0.97-2.40
10-100%	135	1.95	1.36-2.79	2.04	1.35-3.09	1.82	1.25-2.63	2.04	1.33-3.14
**After 2 cycles of therapy, RT-qPCR group**									
negative	48								
<0.1%	133	1.16	0.74-1.80	1.01	0.61-1.68	1.11	0.70-1.75	1.07	0.63-1.81
0.1-1%	45	1.37	0.82-2.31	1.47	0.81-2.64	1.29	0.74-2.24	1.60	0.85-3.00
1-100%	21	2.08	1.14-3.80	2.22	1.16-4.24	1.95	1.02-3.72	2.52	1.24-5.09
**End of induction, RT-qPCR group**									
negative	53								
<0.1%	65	2.30	1.38-3.82	1.85	1.05-3.26	1.94	1.16-3.26	1.60	0.90-2.84
0.1-100%	9	5.38	2.38-12.17	4.55	1.89-10.95	4.40	1.92-10.08	4.00	1.63-9.78
negative	53								
positive	74	2.50	1.52-4.11	2.05	1.18-3.55	2.10	1.27-3.49	1.76	1.01-3.08
**Diagnosis, immunocytology group**									
negative	34								
<10%	140	0.70	0,43-1,14	0.83	0,47-1,46	0.65	0,40-1,07	0.80	0,45-1,42
>10%	127	1.28	0,80-2,05	1.43	0,83-2,49	1.12	0,69-1,82	1.35	0,77-2,39
**After 2 cycles of therapy, immunocytology group**									
negative	86								
<1%	125	0.95	0.67-1.34	0.97	0.65-1.45	0.95	0.66-1.37	0.99	0.65-1.51
>1%	10	2.39	1.21-4.71	3.68	1.83-7.40	2.19	1.11-4.33	3.48	1.72-7.06
**End of induction, immunocytology group**									
negative	80								
positive	23	1.22	0.70-2.15	1.21	0.65-2.25	1.22	0.68-2.19	1.26	0.54-2.42
**Diagnosis, immunocytology negative**									
RT-qPCR negative	14								
RT-qPCR positive	19	1.97	0.75-5.16	3.06	0.85-11.02	1.10	0.36-3.39	1.92	0.49-7.50
**End of induction, immunocytology negative**									
RT-qPCR negative	43								
RT-qPCR positive	37	2.45	1.38-4.34	2.16	1.15-4.06	2.05	1.14-367	1.83	0.97-3.45
**MYCN status**									
not amplified									
amplified		0.99	0.75-1.30	1.14	0.84-1.55				
**Age at diagnosis**									
< 18 months									
> 18 months		1.95	1.27-2.99	1.56	0.99-2.45				
**Patients > 18 months and metastatic disease only**									
** Diagnosis, RT-qPCR group**									
<0.1%	49								
0.1-10%	75	0.94	0,59-1,48	1.09	0,64-1,87	0.94	0,59-1,48	1.08	0,63-1,86
10-100%	128	1.39	0,92-2,09	1.61	0,99-2,60	1.39	0,92-2,10	1.63	1,01-2,64
**After 2 cycles of therapy, RT-qPCR group**									
negative	30								
<0.1%	116	0.9	0,56-1,46	0.89	0,50-1,58	0.87	0,53-1,42	0.93	0,52-1,67
0.1-1%	44	1.02	0,59-1,78	1.24	0,65-2,35	1.02	0,57-1,80	1.39	0,72-2,72
1-100%	21	1.5	0,80-2,81	1.83	0,91-3,67	1.51	0,78-2,90	2.16	1,04-4,51
**End of induction, RT-qPCR group**									
negative	39								
<0.1%	59	1.70	1,01-2,87	1.47	0,82-2,62	1.73	1,03-2,93	1.56	0,87-2,81
0.1-100%	9	3.88	1,71-8,81	3.40	1,40-8,24	4.05	1,77-9,25	3.93	1,60-9,65
negative	39								
positive	68	1.86	1,12-3,10	1.62	0,92-2,85	1.89	1,14-3,16	1.73	0,98-3,06
**Diagnosis, immunocytology group**									
negative	17								
<10%	103	0.34	0,19-0,59	0.39	0,21-0,74	0.36	0,19-0,59	0.38	0,20-0,72
>10%	120	0.55	0,32-0,95	0.66	0,36-1,22	0.55	0,32-0,95	0.65	0,35-1,21
**After 2 cycles of therapy, immunocytology group**									
negative	64								
<1%	113	0.92	0,64-1,34	0.99	0,64-1,52	0.94	0,65-1,38	1.03	0,66-1,60
>1%	10	2.15	1,08-4,28	3.52	1,72-7,21	2.17	1,09-4,32	3.60	1,75-7,39
**End of induction, immunocytology group**									
negative	70								
positive	20	1.09	0,60-1,98	1.03	0,53-2,01	1.15	0,63-2,10	1.15	0,58-2,27
**Landmark analysis**									
** Relapse/progressive disease in first 2 years**									
no									
yes				7.79	4.81-12.61				
**Landmark analysis, 2 years after sample taken, RT-qPCR group at diagnosis**^**a**^									
<0.1%	69								
0.1-10%	77							2.42	1.13-5.19
10-100%	88							2.76	1.32-5.77
**Landmark analysis, 2 years after sample taken, RT-qPCR group after 2 cycles of therapy**^**a**^									
negative	34								
<0.1%	102							1.08	0.44-2.64
0.1-1%	31							1.75	0.64-4.76
1-100%	13							3.32	1.17-9.42
								8.51	4.79-15.12

Hazard Ratio (HR) and 95% confidence intervals (CI) of univariate and multivariate Cox model analyses are indicated for levels of bone marrow infiltration by RT-qPCR or GD2-immunocytology. In multivariate analysis, MYCN status and age >18 months are included as variables

^a^In the landmark analysis, relapse or progressive disease within 2 years after sample collection was included as a variable, MYCN status and age were no variables in this analysis

### Prognostic value of GD2-immunocytology level in BM at diagnosis

**Fig 3:**
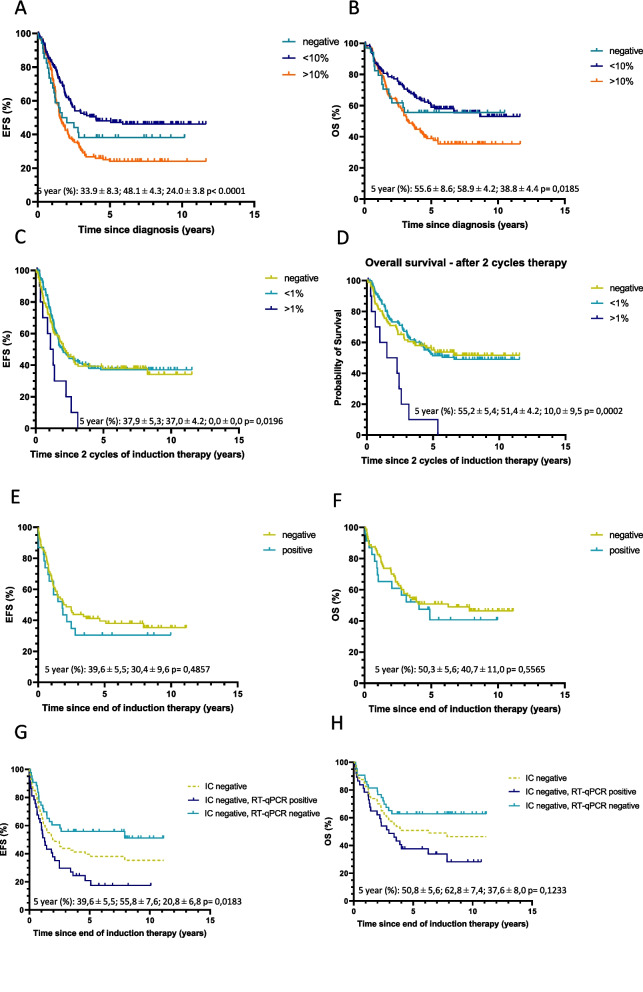
Kaplan-Meier event-free and overall survival curves (EFS on the left and OS on the right, respectively) according to the level of GD2- immunocytology (IC) detected in bone marrow at diagnosis (**A, B**), after 2 cycles of therapy (**C, D**) and after induction therapy (**E, F**). Patients with GD2-immunocytology negative bone marrow at the end of induction therapy, stratified for RT-qPCR results (**G, H**). 5-year survival rates are given in order according to the legend. The number of children at risk with time for each group is provided in the Data Supplement

For immunocytology, the level of infiltration was negatively associated with outcome (Fig 3A-B), however, it did not differ in terms of EFS and OS from immunocytology negative patients (Table 2, Supplemental Figure 6 A-B), possibly due to the relative poor outcome of those patients with immunocytology negative BM. Within the small cohort of 33 immunocytology negative samples (for 1 sample no material was available for RT-qPCR), a positive RT-qPCR appeared to predict a worse prognosis, however this difference did not reach significance (Table 2; Supplemental Figure 6C-D). In patients older than 18 months with metastatic disease, patients with GD-2 negative BM had a remarkably poor prognosis (Supplemental Figure 4G-H, Table 2).

### Clinical significance of BM infiltration after two induction therapy courses

We previously showed in a retrospective cohort that fast-response correlated with better outcome. To study prospectively the clinical significance of early BM clearance, BM infiltration was analyzed by RT-qPCR after 2 induction therapy courses (n=247). Fast response after two therapy courses was associated with survival, a significantly poor prognosis was only seen for children with RT-qPCR BM infiltration >1% (adjusted HR 1.95 [95% CI 1.02-3.72] and 2.52 [1.24‐5.09] for EFS and OS, respectively) (Figure 2C-D, Table 2). This was also shown in the subgroup analysis of patients with metastatic disease, older than 18 months (Supplemental Figure 4C-D, Table 2) and in the landmark analysis starting 2 years after the sample was taken, (HR for OS RT-qPCR 0.1-1% 3.32 [1.17-9.42] and RT-qPCR>1% 8.51 [4.79-15.12]) (Table 2, Supplemental Figure 5D-F). Immunocytology after 2 cycles of therapy also confirmed the poor prognostic value of >1% BM infiltration, with a 5-year EFS of 0% versus 37.9 (5.3) and OS of 10% (9.5) versus 55.2 (5.4), also in the cohort of patients older than 18 months with metastatic disease (Table 2, Figure 3C-D, Supplemental Figure 4I-J).

### Clinical significance of BM clearance at the end of induction therapy

To validate the correlation of BM response with outcome, we tested both RT-qPCR and immunocytology at end of induction therapy (median 184 days since diagnosis). In 74 out 127 patients neuroblastoma mRNA was still detected (poor-responders), which correlated with poor outcome: 5-years EFS was 26.6% (5.2) versus 60.4% (6.7) and OS was 43.8% (5.9) versus 65.7% (6.6) for RT-qPCR positive patients versus RT-qPCR negative patients (Figure 2E-H, Supplemental Table 1). Compared to the outcome of the whole cohort, any RT-qPCR-positivity correlated with poorer outcome (Figures 2G-H). In multivariate Cox regression model, end of induction RT-qPCR positivity was associated with poor EFS and OS, with a HR of 2.10 [1.27-3.49] and 1.76 [1.01-3.08] respectively (Table 2), also for the patients with metastatic disease, older than 18 months (Supplemental Figure 4E-F, Table 2). In contrast, end of induction immunocytology positivity was not associated with EFS or OS (HR 1.22 [0.68-2.19] and 1.26 [0.54-2.42]) (Table 2, Figure 3E-F), neither in the total high-risk cohort, nor in the subgroup of patients older than 18 months with metastatic disease only. Moreover, because we analyzed RT-qPCR and immunocytology in the same samples, we could classify the immunocytology-negative samples according to their RT-qPCR score. This clearly resulted in a significantly poorer outcome for the immunocytology-negative/RT-qPCR positive group, versus negative for both techniques (univariable HR 2.45 [1.38-4.35] and 2.16 [1.15-4.06] for EFS and OS respectively, and 5-year EFS of 20.8 (6.8) versus 55.8 (7.6) and 5-year OS of 37.6 (8.0) versus 62.8 (7.4)). Difference in survival in this immunocytology negative group remains significant in multivariate analysis for EFS (HR 2.05 [1.14-3.67]) (Table 2, Figure 3G-H). Immunocytology positive results correspond mainly to RT-qPCR positive BM, with only 2 immunocytology positive/RT-qPCR negative BM samples (Supplemental Figure 6E-F). We conclude that at the end of induction therapy, any RT-qPCR positivity, in contrast to immunocytology, identifies patients within the high-risk cohort with a very poor outcome.

### Contribution of the different RT-qPCR markers

**Fig 4:**
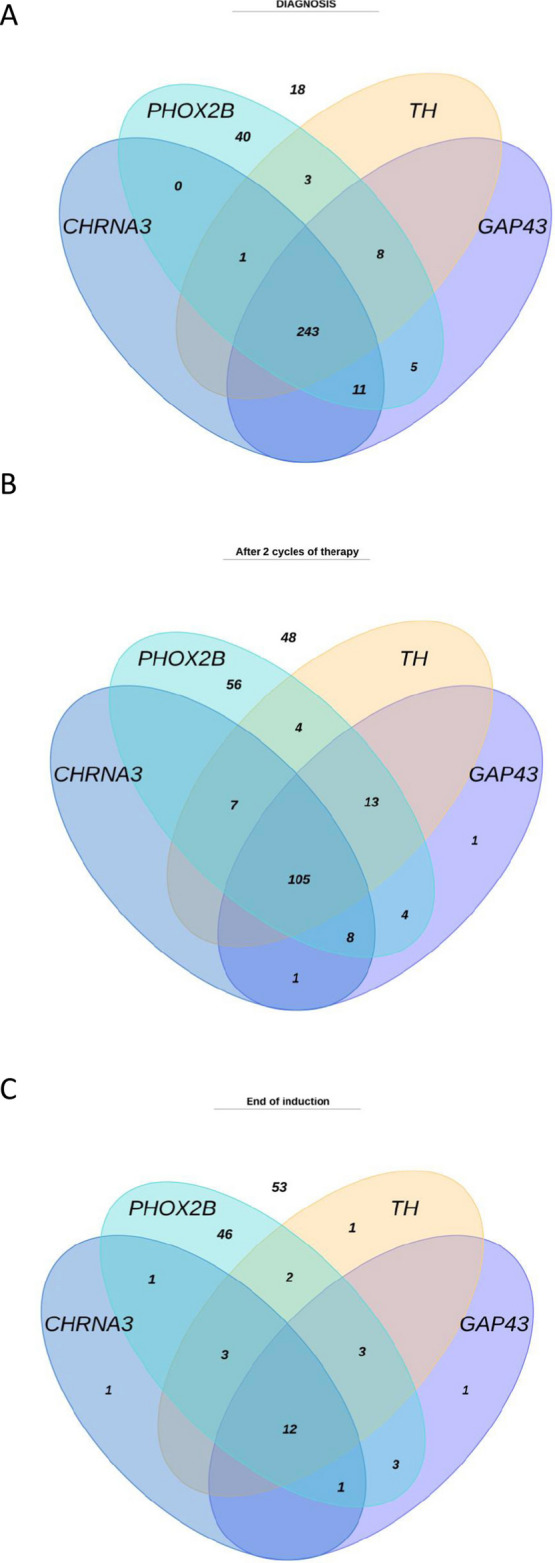
Contribution of the different mRNA markers to the positive samples at (**A**) diagnosis; (**B**) after 2 cycles of therapy and (**C**) end of induction therapy. Each ellipse represents positive results of one marker. The number of RT-qPCR negative samples are stated in white on top of the diagram

All samples collected at three different timepoints were included in this analysis. During an interim analysis in 2014 [35] DDC was the least informative marker and has therefore been excluded for further testing since then. At diagnosis, 311/329 samples (95%) showed positive results for one or more markers with 74% being positive for all markers (Figure 4A). *PHOX2B* was the most sensitive marker and was positive in 100% of positive diagnostic samples. In the follow-up samples, different markers/marker-combinations contributed to positivity (Figure 4B-C), again with *PHOX2B* positivity most frequently observed (80% after 2 therapy cycles and 56% at end of induction). Of the positive samples, only 1.0% after 2 cycles and 2.4% at end of induction were *PHOX2B-*negative. Supplemental Figures 7-9 and Supplemental Table 2 show the prognostic value of each individual marker. *PHOX2B* positivity at end of induction was predictive of EFS and OS in univariate analysis, similar to *DDC*, *CHRNA3* and *GAP43*. Nevertheless, none of the individual markers, except for DDC, which was only performed in a small cohort, was more predictive of outcome compared to the combined RT-qPCR panel.

## Discussion

While a great portion of high-risk neuroblastoma patients achieve remission at the end of first line treatment, unfortunately more than half of these patients still experience relapse. MRD in the BM is thought to be the leading cause of relapse, even after intensive treatment [6]. Early MRD detection is necessary for disease monitoring and predicting therapy response to achieve optimal outcome [36, 37]. GD2-immunocytology is recommended for BM evaluation by the INRC BM working group [5, 8], and is shown to be more sensitive than conventional cytology [13], as was RT-qPCR [21]. We show in one of the largest published prospective high-risk cohorts the prognostic value of neuroblastoma mRNA detection in BM at diagnosis, during, and at end of induction therapy, and compare these with the current diagnostic standard for BM infiltration, GD2-immunocytology. We show that RT-qPCR detecting our panel of neuroblastoma mRNA markers is an independent predictor of outcome. Very sensitive BM MRD detection by RT-qPCR for our mRNA panel is superior to GD2-immunocytology, not only in terms of prognostic value but also for sensitive and reliable detection of BM clearance. At diagnosis we already identify a subgroup (41%) with BM infiltration >10% that are at high-risk for suffering from relapse or progressive disease. At end of induction therapy, when patients proceed to consolidation therapy, neuroblastoma mRNA RT-qPCR identifies a large group (58%) which still has BM infiltration and poor prognosis. End of induction GD2-immunocytology results did not correlate with outcome. Strikingly, RT-qPCR stratified the immunocytology negative patients in those with truly negative BM and those with still detectable MRD. These findings show that RT-qPCR has superior sensitivity, and that low levels of BM disease, detected only by RT-qPCR, correlate with poor outcome. This is in line with other studies from Viprey, Träger and Yáñez [17, 38, 39]. For patients within this high-risk group novel treatment options should be discussed, e.g. tandem autologous stem cell transplant [40], promising chemo-immunotherapies [41, 42] or precision medicine programs such as the INFORM Registry [43], and evaluated in clinical trials. Studies should furthermore investigate if these are the patients with SIOPEN >3 skeletal scores who have a poorer outcome [44–46], and if alternative therapy approaches, evaluated in clinical trials, can rescue these patients. Our study furthermore demonstrates the robustness of RT-qPCR mRNA detection, as it validates the clinical significance of neuroblastoma-specific mRNA detection in BM in a different high-risk chemotherapy background, and using a different marker panel and cut points then Viprey et al [17].

Multiple studies made use of distinct marker panels and different methods for defining a threshold for positivity of MRD [15, 17, 18, 21, 47–49]. Viprey et al. defined a threshold for positivity by calculating a cut point for mRNA based on a test cohort that is associated with survival [17]. Efforts to validate both the SIOPEN and DCOG/GPOH approach are ongoing. To avoid false-positive results, we base our thresholds for positivity on expression of the markers in normal control BM samples [20]. Furthermore, these thresholds are not dependent on changes in treatment protocols. Although in our study we estimate BM infiltration levels by relating the expression levels of the mRNA panel to that in the neuroblastoma cell line IMR-32, the estimated infiltration percentage by RT-qPCR, which are mRNA transcripts, overall corresponds to the infiltration by GD2-immunocytology. Several MRD markers were tested in the search for optimal markers in neuroblastoma over the past years. Due to the heterogeneous expression of marker genes amongst patients, and within an individual tumor and its metastasis, the use of a marker panel is superior to using a single marker [21, 22, 47, 50, 51]. In our study, *PHOX2B* is the most specific and sensitive marker. It should be noted that this *PHOX2B* assay, developed by Stutterheim et al., is different from the one used by Viprey and colleagues [17, 20]. During an interim analysis in 2014 [35], *DDC* was the least informative marker, being solely positive in only one follow-up sample and was subsequently excluded. As none of the markers contributed individually more to the outcome than the whole panel, we propose to use the panel of four markers (*PHOX2B*, *TH*, *CHRNA3*, *GAP43*). Although this panel of markers is superior to current techniques to detect neuroblastoma presence in the BM, we unfortunately still see a group of patients, free from BM infiltration, who still suffer from recurrent disease. It might be that the residual tumor cells causing the relapse do not reside in the BM, or the tumor cells residing in the BM escape surveillance due to a low expression of the used MRD markers. The commonly used MRD markers are selected on expression levels in primary tumors and cell lines, but not on treated tumors. Epithelial-to-mesenchymal transition (EMT)—the process by which epithelial cells transform to a mesenchymal phenotype—is associated with tumor progression, metastasis, and therapy resistance in several cancer types [52]. The process of EMT, or in case of neuroblastoma, adrenergic to mesenchymal transition, has also been demonstrated to generate cellular heterogeneity in neuroblastoma [53, 54]. We confirmed that the commonly used neuroblastoma MRD markers (including *PHOX2B, TH, CHRNA3, GAP43*) are rarely expressed in mesenchymal neuroblastoma cell lines. We therefore identified a panel of markers, specific for the detection of the mesenchymal neuroblastoma cells [55]. In a follow-up study, we are currently investigating the clinical significance of these markers in the same cohort used in this study. As the number of MRD mRNA markers is expanding with the addition of these markers, we developed a reliable and sensitive multiplex panel for (adrenergic and mesenchymal) MRD markers, after completing this prospective study, to save time, make optimal use of these precious samples, and to facilitate the use of a panel of mRNA markers in the clinic [51]. This study has two main limitations, which also are present in studies of Viprey and Yáñez [17, 39]. Similar to other studies, the first limitation is the number of missing samples during and at end of induction therapy. This was mostly the result of logistical or clinical failures, previous GD2-immunocytology results, which were reported back to the treating physician, did not influence subsequent sampling. The second limitation is the fact that in the rare disease neuroblastoma, international prospective studies take many years to complete, so these often lack prospectively reported data on other disease evaluation modalities such as MIBG/imaging scans or urine catecholamines. Marachelian et al. already showed the additional value of neuroblastoma mRNA for disease evaluation compared to MIBG and BM morphology in cohort with relapsed/refractory patients [47]. These data will be prospectively collected in the SIOPEN High-Risk Neuroblastoma 2 trial (ClinicalTrials.gov Identifier: CT04221035). In this trial, we will also analyze the value of bilateral bone marrow sampling versus single site sampling only by comparing highest, lowest and median infiltration. In this cohort, this was not possible as most samples (all German samples) were pooled before analysis [7, 41].

MRD analysis by RT-qPCR has several advantages: quantification can be reliably performed [22], it is relatively inexpensive, less dependent on quality of smears and not dependent on interobserver variability or experience of the investigator, compared to cytology & immunocytology [32]. While the emerging technique droplet digital PCR (ddPCR) can robustly quantify low levels of tumor derived nucleic acids with high precision, it is less suited for materials with high RNA or DNA content, such as the bone marrow. (RT-)qPCR is very well suited for MRD detection in a broad dynamic range, such as the expression levels of neuroblastoma mRNA in BM [56]. The use of cell-free DNA (cfDNA) in liquid biopsies is successfully being studied in adult cancers, and starting to emerge in pediatric cancers. The question can arise if mRNA or cfDNA is more optimal for MRD detection. Our studies in neuroblastoma have shown not only the concordance but also the discordance when testing both our mRNA-panel as well as circulating tumor-derived DNA in paired samples, demonstrating the need to implement both techniques as future clinical test [57, 58].

## Conclusion

In this prospective study, we show the clinical relevance of MRD detection by RT-qPCR in neuroblastoma. The mRNA panel currently studied shows a strong association between BM infiltration levels and EFS and OS at different time-points. Any end of induction BM positivity by RT-qPCR is significantly associated with poor survival and identifies patients at risk for relapse. Molecular detection of MRD by RT-qPCR was more sensitive and of higher prognostic value than immunocytology for neuroblastoma BM infiltration. Thus, we suggest the implementation of MRD detection by RT-qPCR in clinical practice.

## Supplementary Information

Supplementary Material 1: Supplemental Figure 1. Schematic overview of Dutch (DCOG NBL2009 trial) and German (GPOH NB2004-HR) trials. Dutch patients received 2 upfront courses of MIBG therapy, if clinically achievable. German patients were randomized to standard induction therapy, or two additional courses of N8 upfront. N5 = vindesine, cisplatin, etoposide; N6= vincristine, dacarbacine, ifosfamide, doxorubicin; N8 = topotecan, cyclophosphamide and etoposide; HD chemo = melphalan, carboplatin, etoposide, followed by autologous stem cell transplantation; RTx= radiotherapy, IT= immune therapy. Blue arrows indicate time points of sample acquisition. Supplemental Figure 2. Sample consort diagram. We investigated whether previous negative immunocytology results influenced sample acquisition. Of the 88 missing bone marrow samples after 2 cycles of therapy, 23 (26%) samples were from patients with a previous sample negative for immunocytology, while of the 257 sampled patients after 2 cycles of therapy, only 30 patients did have a negative previous sample or were not sampled before (12%). Of the 216 patients with missing samples at the end of induction, 102 patients (47%) had a previously negative sample for immunocytology, similarly to the 129 patient that were sampled at the end of induction (61 patients with a previously negative sample; 47%). Supplemental Figure 3 (A) Number of samples grouped by infiltration by RT-qPCR at diagnosis, after 2 cycles of therapy (2 CT) and at the end of induction therapy. (B) Number of samples grouped by infiltration based on GD2-immunocytology at diagnosis, after 2 cycles of therapy (2 CT) and at the end of induction therapy. Supplemental Figure 4. Kaplan-Meier event-free and overall survival curves (EFS on the left and OS on the right, respectively) of the cohort with patients stage M and age >18 months, according to the level of mRNA infiltration by RT-qPCR detected in bone marrow at diagnosis (A, B), after 2 cycles of therapy (C, D), after induction therapy (E, F) and according to the level of GD2- immunocytology (IC) detected in bone marrow at diagnosis (G,H), after 2 cycles of therapy (I,J) and after induction therapy (K,L). 5-year survival rates are given in order according to the legend. The number of children at risk with time for each group is provided in the Data Supplement. Supplemental Figure 5. Landmark Kaplan-Meier overall surival (OS) analysis, starting 2 years after sample collection for A. diagnosis, stratified for relapse or progressive disease in the first 2 years after sample collection; B. level of bone marrow infiltration by RT-qPCR at diagnosis; C. diagnosis, stratified for RT-qPCR group and relapse/progressive disease; D. after 2 cycles of therapy, stratified for relapse or progressive disease in the first 2 years after sample collection; E. level of bone marrow infiltration by RT-qPCR after 2 cycles of therapy; F. after 2 cycles of therapy, stratified for RT-qPCR group and relapse/progressive disease. 5-year survival rates are given in order according to the legend.The number of children at risk with time for each group is provided in the Data Supplement. Supplemental Figure 6. Kaplan-Meier event-free and overall survival curves (EFS on the left and OS on the right, respectively) according to the level of GD2- immunocytology (IC) detected in bone marrow at diagnosis (A, B). Kaplan-Meier curves for patients with GD-2 IC negative bone marrow at diagnosis, stratified for RT-qPCR results (C, D) and patients with GD2- immunocytology positive bone marrow at the end of induction, stratified for RT-qPCR results (E, F). 5-year survival rates are given in order according to the legend. The number of children at risk with time for each group is provided in the Data Supplement. Supplemental Figure 7. Kaplan-Meier event-free and overall survival curves (EFS on the left and OS on the right, respectively) according to individual marker positivity in bone marrow samples at diagnosis. If a marker had a Ct value<40 but ΔCt of the sample was within 3 Ct of the ΔCt of the normal control BM samples, it was regarded as negative for analysis, but is depicted here as‘below threshold’. 5-year survival rates are given in order according to the legend. The number of children at risk with time for each group is provided in the Data Supplement. Supplemental Figure 8. Kaplan-Meier event-free and overall curves (EFS on the left and OS on the right, respectively) according to individual marker positivity in bone marrow samples after 2 cycles of therapy. If a marker had a Ct value<40 but ΔCt of the sample was within 3 Ct of the ΔCt of the normal control BM samples, it was regarded as negative for analysis, but is depicted here as ‘below threshold’. 5-year survival rates are given in order according to the legend. The number of children at risk with time for each group is provided in the Data Supplement. Supplemental Figure 9. Kaplan-Meier event-free and overall curves (EFS on the left and OS on the right, respectively) according to individual marker positivity in bone marrow samples at the end of induction therapy. If a marker had a Ct value<40 but ΔCt of the sample was within 3 Ct of the ΔCt of the normal control BM samples, it was regarded as negative for analysis, but is depicted here as ‘below threshold’. 5-year survival rates are given in order according to the legend. The number of children at risk with time for each group is provided in the Data Supplement.

Supplementary Material 2: Supplemental Table 1. Number of children at risk with time in Kaplan Meier plots shown in Figure 2, 3, Supplemental Figures 4, 5, 6, 7-9.

Supplementary Material 3: Supplemental Table 2. Event-free survival (EFS) and overall survival (OS) in Cox proportional hazards regression models per individual RT-qPCR marker. Hazard Ratio (HR) and 95% confidence intervals (CI) of univariable and multivariable Cox model analyses are indicated for each RT-qPCR marker. In multivariate analysis, RT-qPCR group,MYCN status and age >18 months are included as variables.

## Data Availability

The data during and/or analysed during the current study available from the corresponding author on reasonable request.

## Abbreviations

BM: Bone marrow
cfDNA: Cell-free DNA
CHRNA3: Cholinergic receptor nicotinic alpha 3
DCOG: Dutch Childhood Oncology Group
DCX: Doublecortin
DDC: Dopamine decarboxylase
EFS: Event free survival
GAP43: Growth associated protein 43
GD2: Disialoganglioside GD2
GPOH: Gesellschaft für Pädiatrische Onkologie und Hämatologie
GUSB: Beta-glucoronidase
HR: Hazard Ratio
INRC: International Neuroblastoma Response Criteria
INSS: International Neuroblastoma Staging System
MIBG: Metaiodobenzylguanidine
MRD: Minimal residual disease
MRI: Magnetic resonance imaging
MYCN: N-Myc proto-oncogen
N5: Chemotherapy regimen consisting of vindesine, cisplatin, etoposide
N6: Chemotherapy regimen consisting of vincristine, dacarbacine, ifosfamide, doxorubicin
N8: Chemotherapy regimen consisting of topotecan, cyclophosphamide and etoposide
OS: Overall survival
PB: Peripheral blood
PET: Positron emission tomography
PHOX2B: Paired-like homeobox 2b
RT-qPCR: Reverse transcriptase quantitative polymerase chain reaction
SIOPEN: European Society of Pediatric Oncology Neuroblastoma Group
TH: Tyrosine hydroxylase
