# Supplementary material for: Sensitive liquid biopsy monitoring correlates with outcome in the prospective international GPOH-DCOG high-risk neuroblastoma RT-qPCR validation study
Source: J Exp Clin Cancer Res. 2024 Dec 26;43:331. doi: 10.1186/s13046-024-03261-y (PMC11670466; doi:10.1186/s13046-024-03261-y)
Supplement: Supplementary file 1 — Supplementary Material 1: Supplemental Figure 1. Schematic overview of Dutch (DCOG NBL2009 trial) and German (GPOH NB2004-HR) trials. Dutch patients received 2 upfront courses of MIBG therapy, if clinically achievable. German patients were randomized to standard induction therapy, or two additional courses of N8 upfront. N5 = vindesine, cisplatin, etoposide; N6= vincristine, dacarbacine, ifosfamide, doxorubicin; N8 = topotecan, cyclophosphamide and etoposide; HD chemo = melphalan, carboplatin, etoposide, followed by autologous stem cell transplantation; RTx= radiotherapy, IT= immune therapy. Blue arrows indicate time points of sample acquisition. Supplemental Figure 2. Sample consort diagram. We investigated whether previous negative immunocytology results influenced sample acquisition. Of the 88 missing bone marrow samples after 2 cycles of therapy, 23 (26%) samples were from patients with a previous sample negative for immunocytology, while of the 257 sampled patients after 2 cycles of therapy, only 30 patients did have a negative previous sample or were not sampled before (12%). Of the 216 patients with missing samples at the end of induction, 102 patients (47%) had a previously negative sample for immunocytology, similarly to the 129 patient that were sampled at the end of induction (61 patients with a previously negative sample; 47%). Supplemental Figure 3 (A) Number of samples grouped by infiltration by RT-qPCR at diagnosis, after 2 cycles of therapy (2 CT) and at the end of induction therapy. (B) Number of samples grouped by infiltration based on GD2-immunocytology at diagnosis, after 2 cycles of therapy (2 CT) and at the end of induction therapy. Supplemental Figure 4. Kaplan-Meier event-free and overall survival curves (EFS on the left and OS on the right, respectively) of the cohort with patients stage M and age >18 months, according to the level of mRNA infiltration by RT-qPCR detected in bone marrow at diagnosis (A, B), after 2 cycles of therapy (C, D), [file 13046_2024_3261_MOESM1_ESM.pdf]

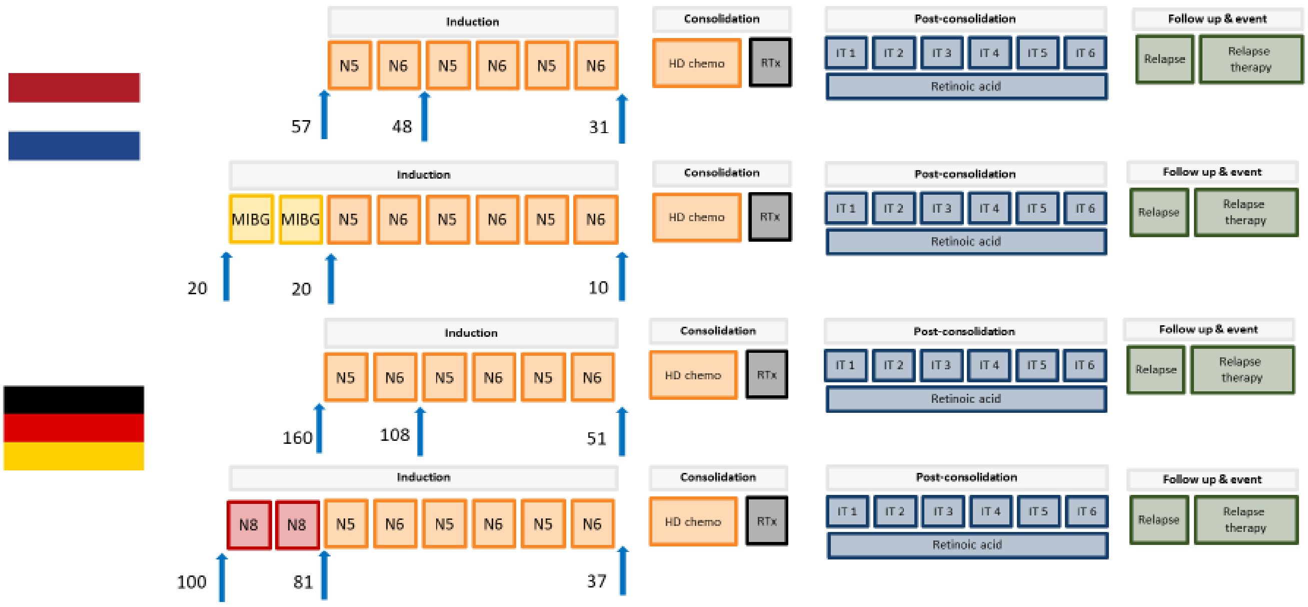

Supplemental Figure 1.

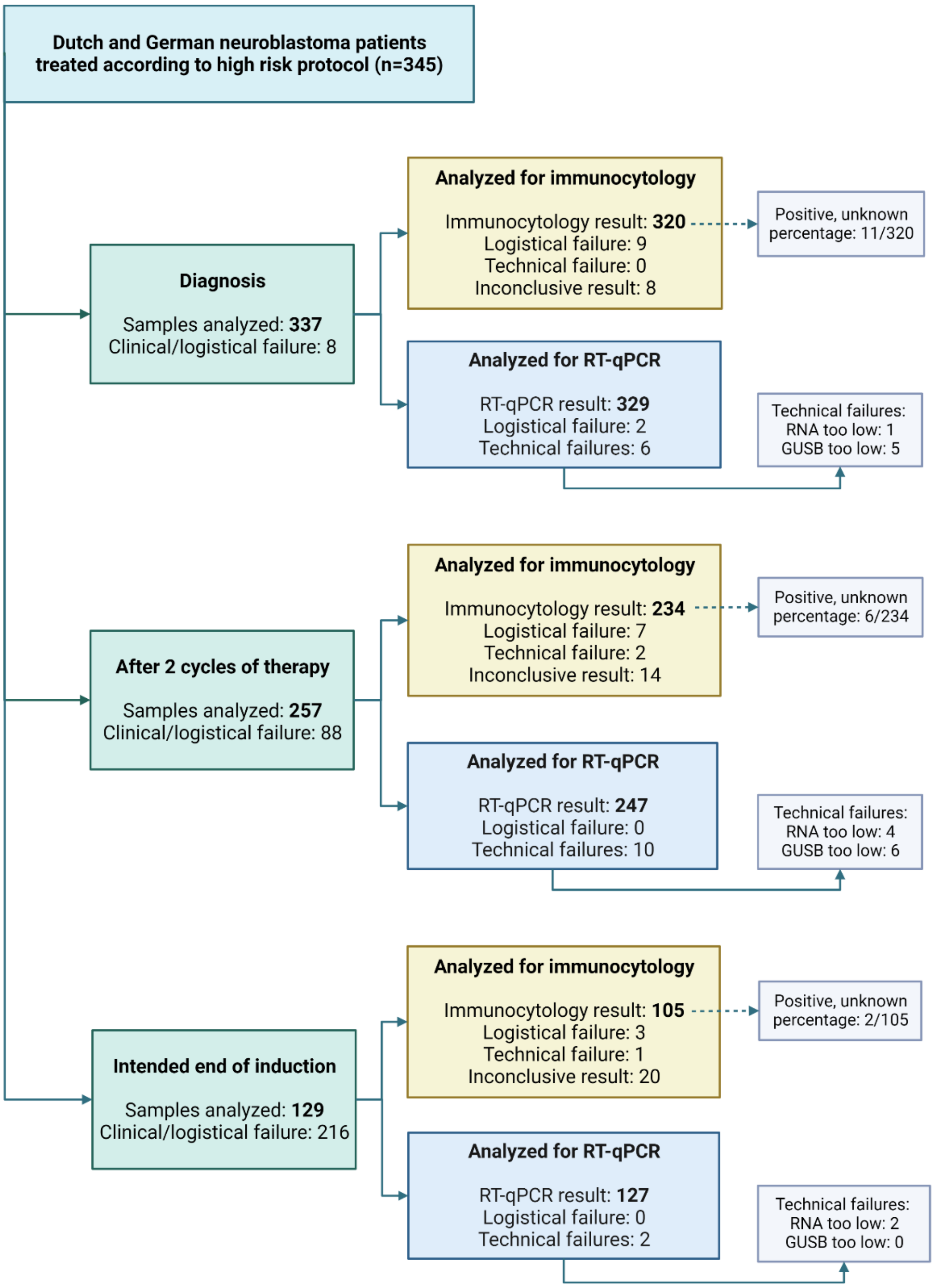

Supplemental Figure 2.

A

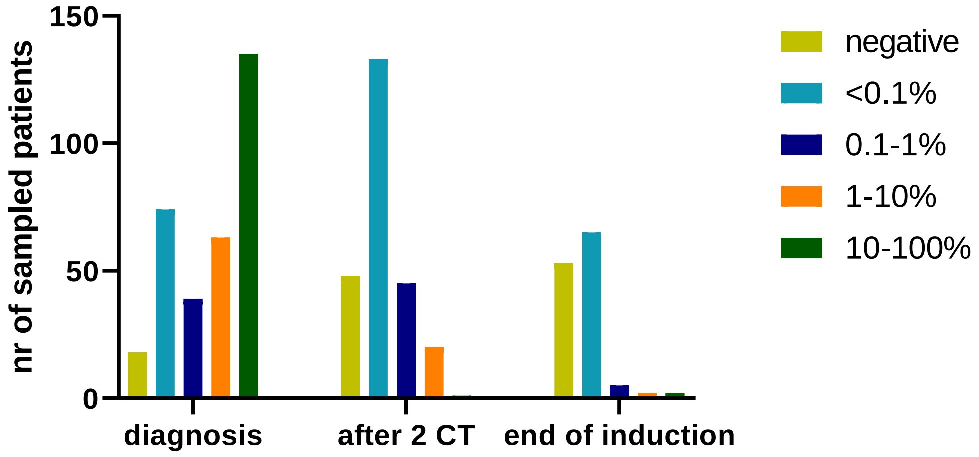

B

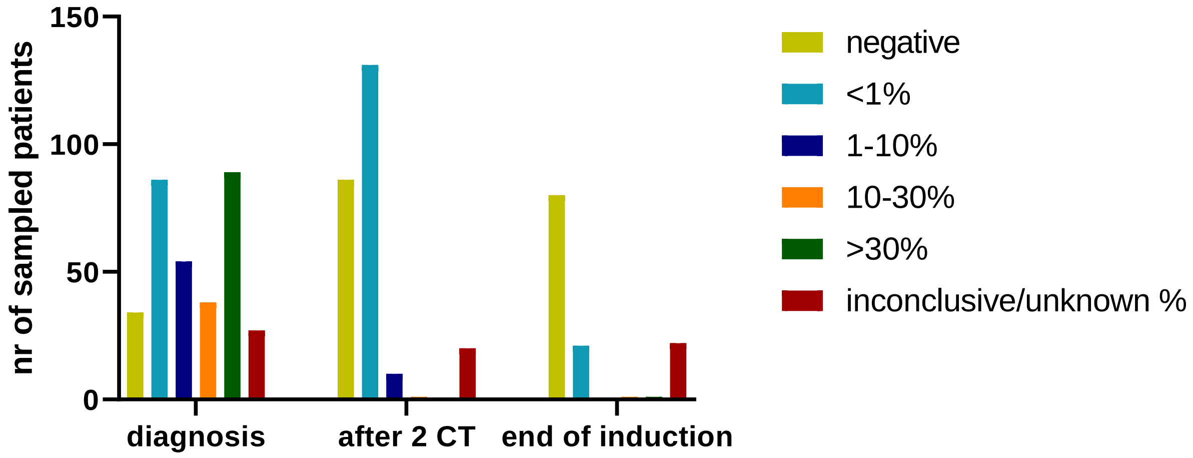

Supplemental Figure 3

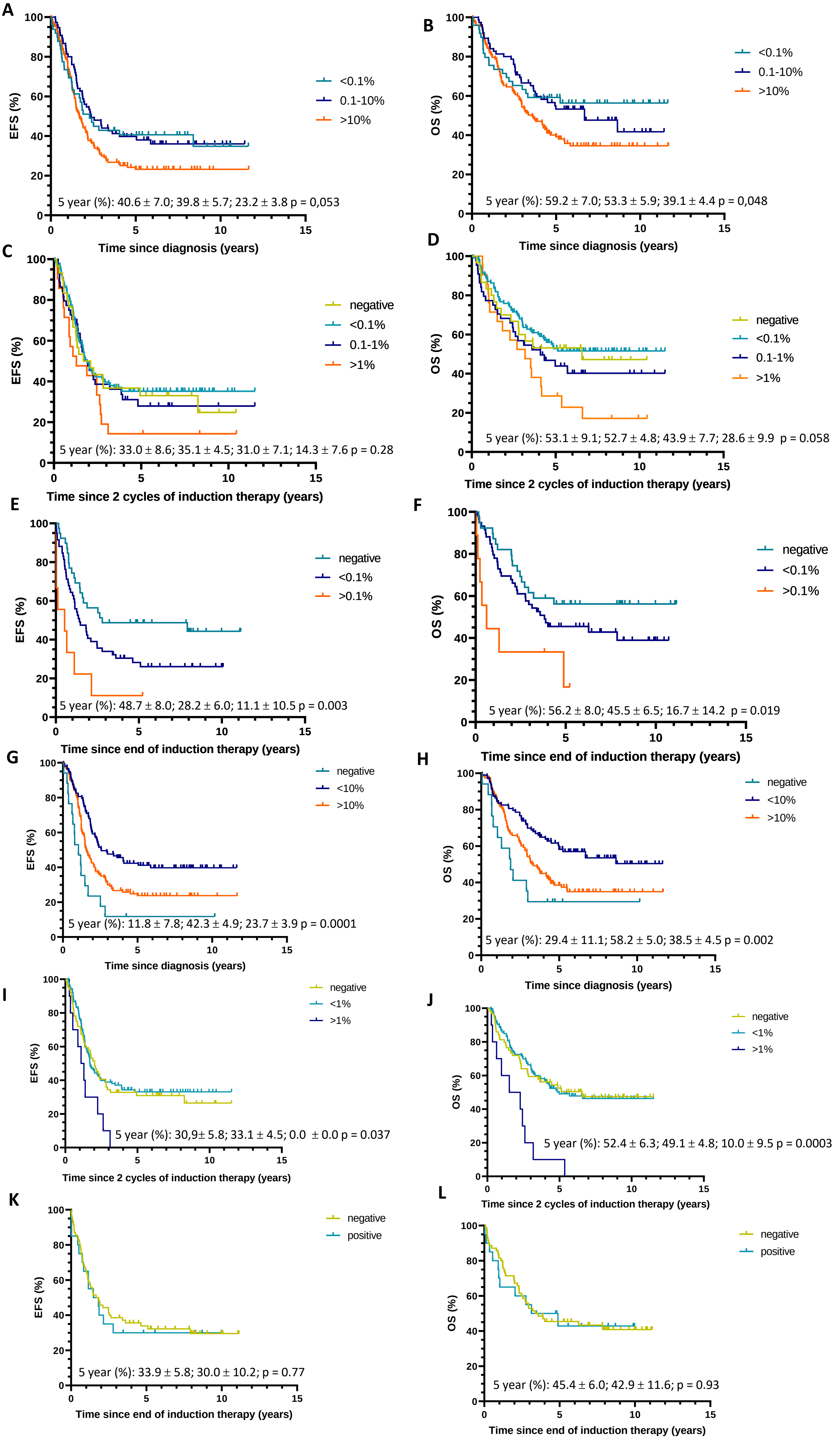

Supplemental Figure 4.

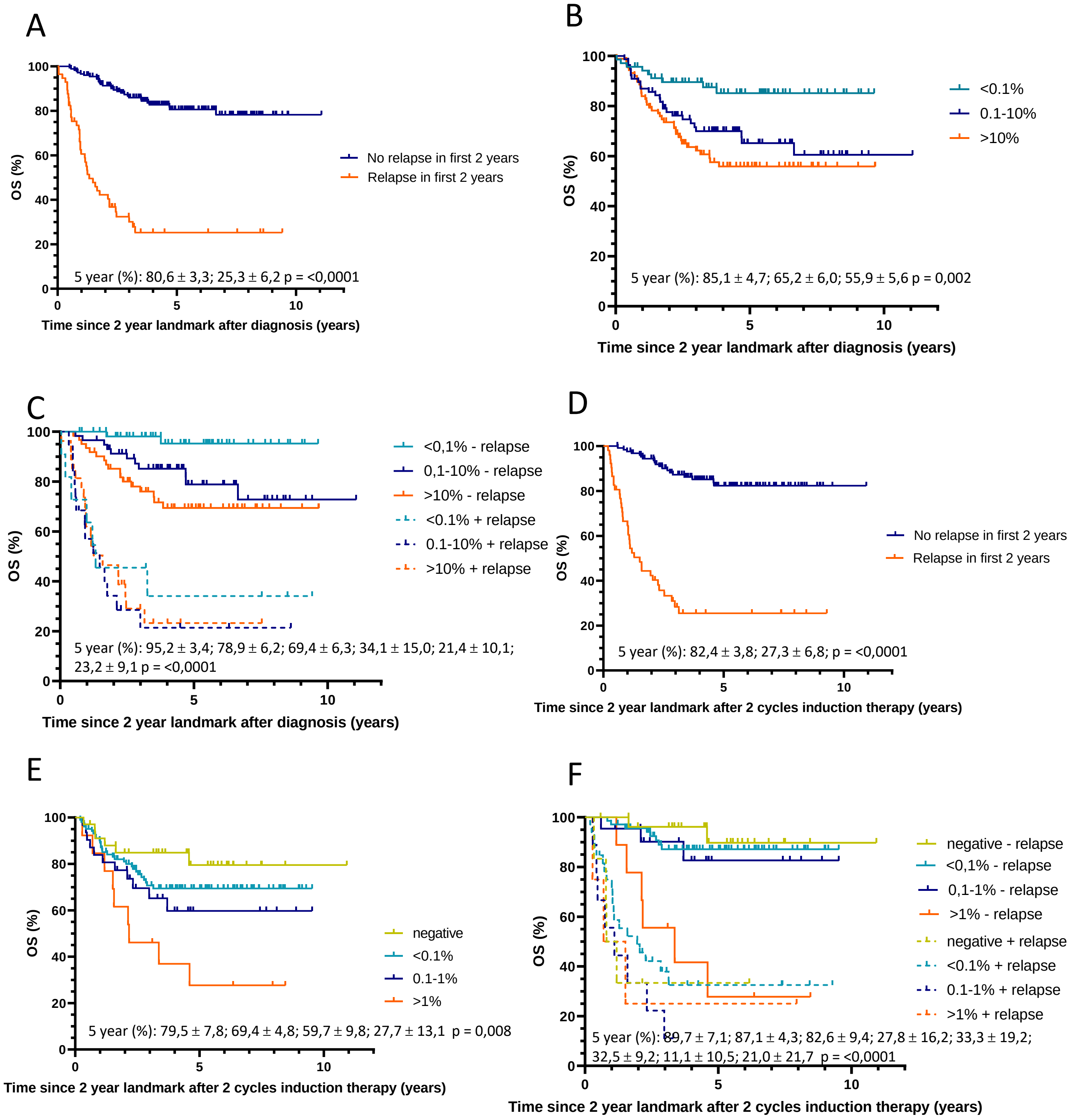

Supplemental Figure 5.

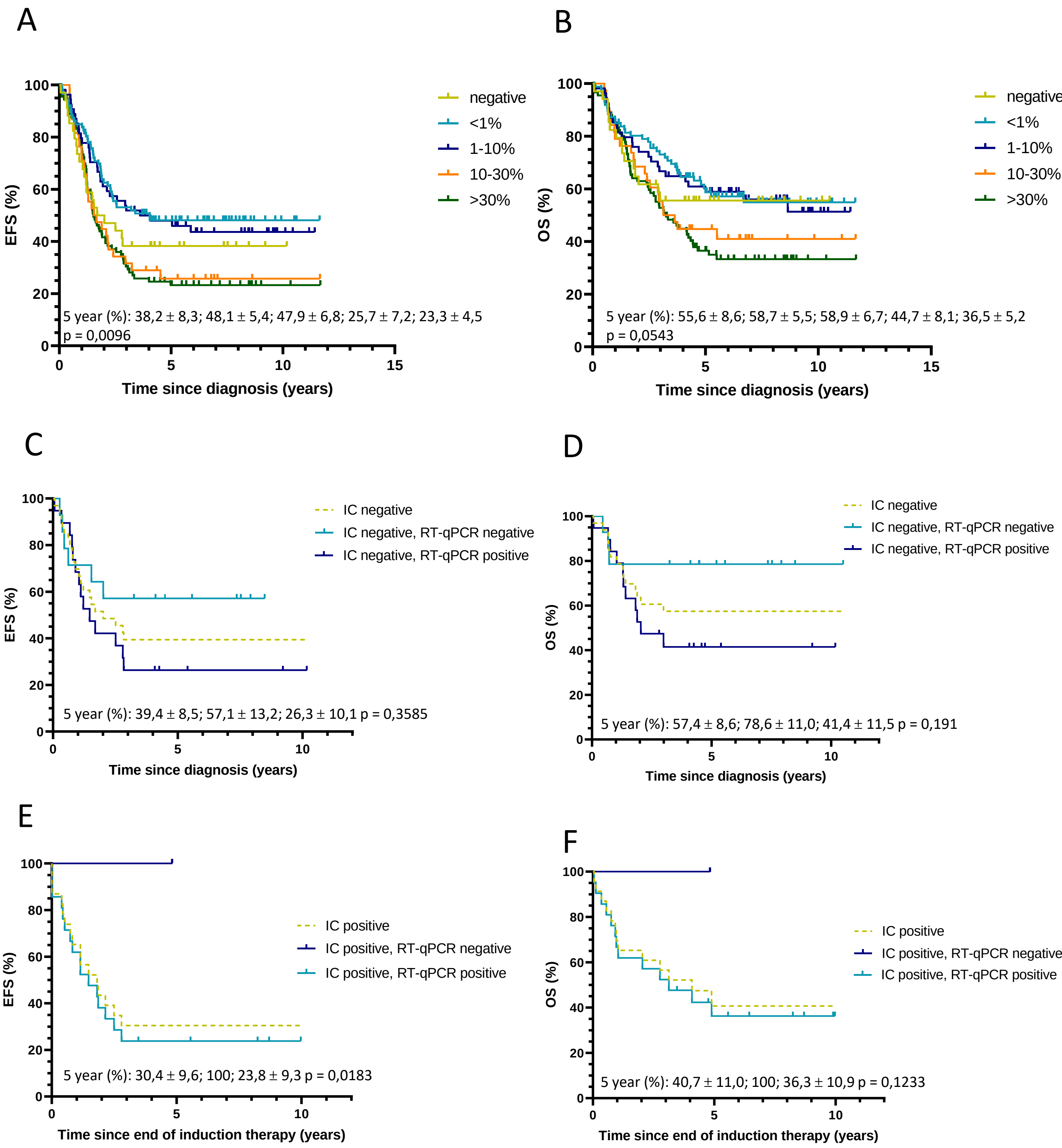

Supplemental Figure 6.

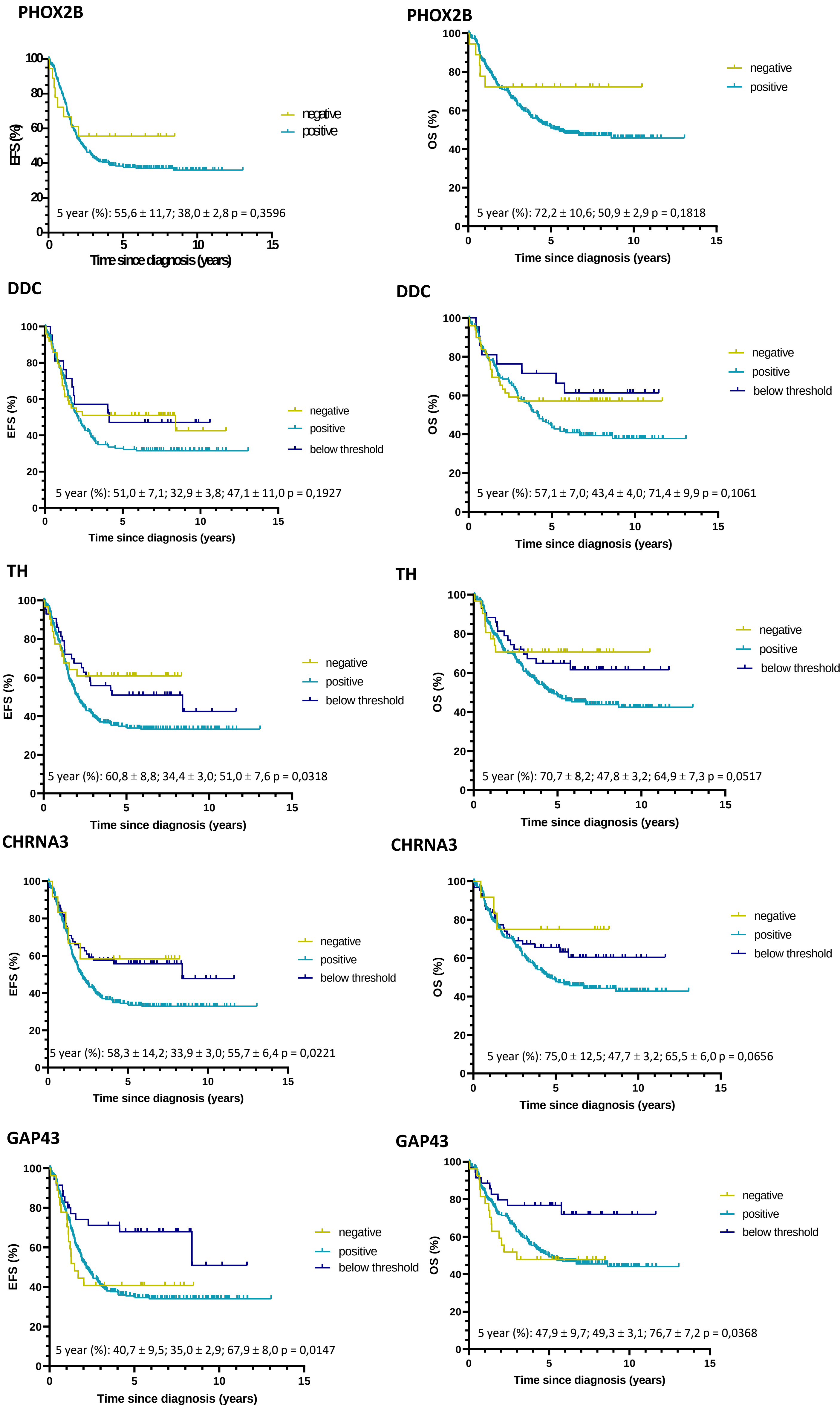

Supplemental Figure 7.

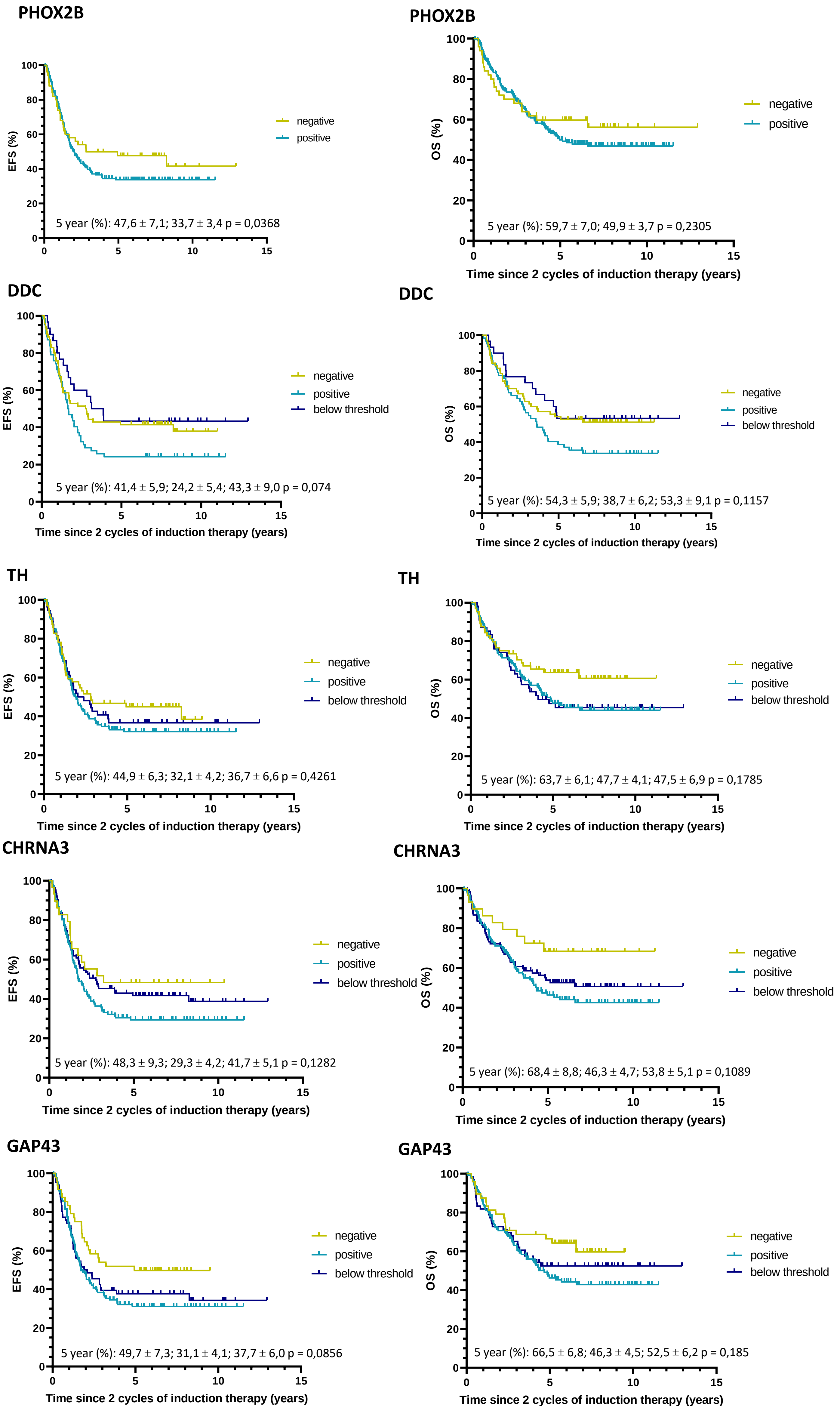

Supplemental Figure 8.

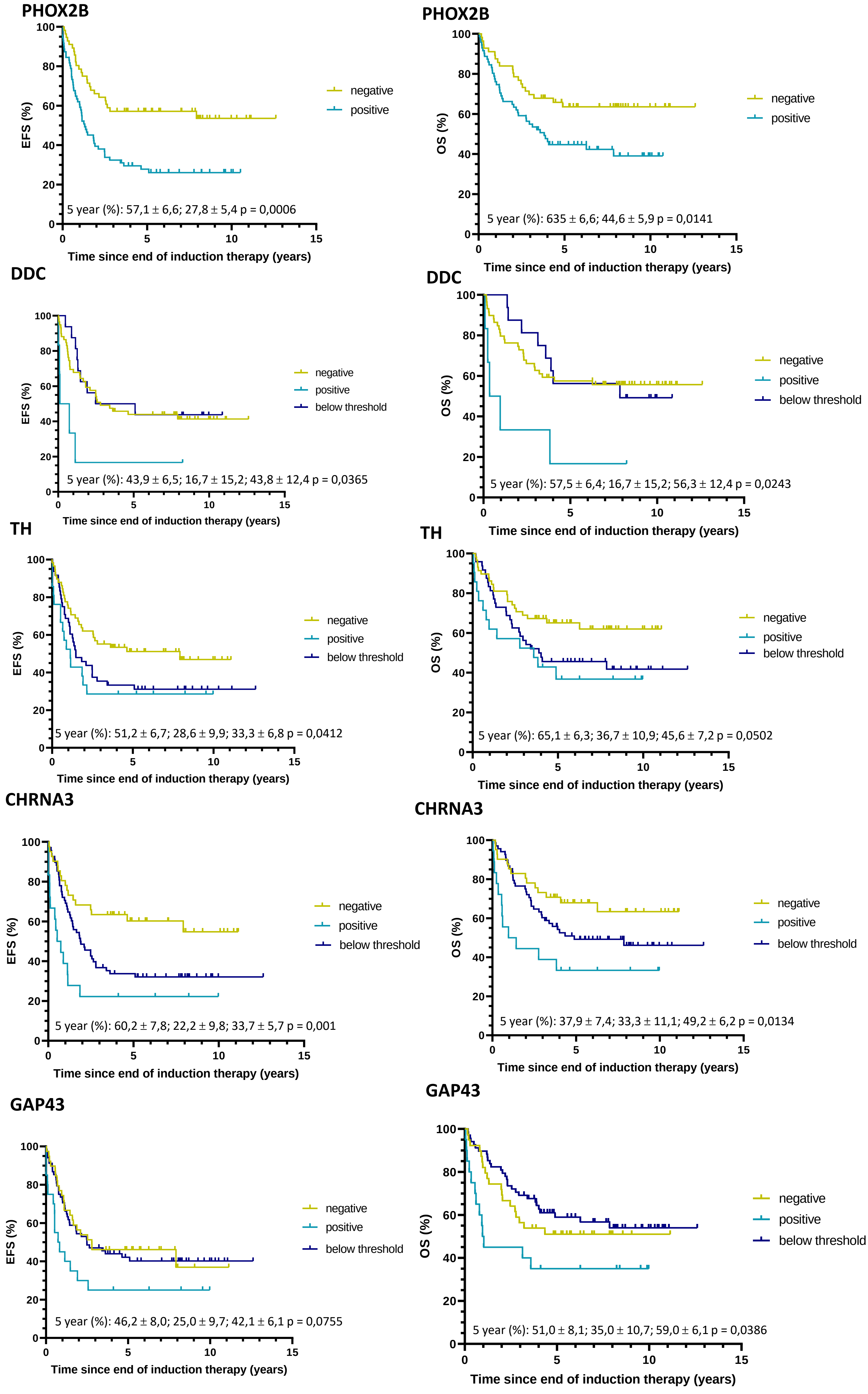

Supplemental Figure 9.
